# Analgesic effect of perioperative duloxetine in patients after total knee arthroplasty: a prospective, randomized, double-blind, placebo-controlled trial

**DOI:** 10.1186/s12891-022-05194-z

**Published:** 2022-03-12

**Authors:** Mingcheng Yuan, Tingting Tang, Zichuan Ding, Hao Li, Zongke Zhou

**Affiliations:** Department of Orthopedics, West China Hospital/West China School of Medicine, Sichuan University, 37# Wuhou Guoxue Road, Chengdu, P.R. China

**Keywords:** Total knee arthroplasty, Duloxetine, Analgesic effect, Randomized controlled trial

## Abstract

**Background:**

To investigate the analgesic effect of perioperative use of duloxetine in patients received total knee arthroplasty (TKA).

**Method:**

This prospective randomized, double-blind, placebo-controlled trial study was registered in the Chinese Clinical Trial Registry (ChiCTR2000033910). 100 patients were finally enrolled. The hospital pharmacy prepared small capsules containing either duloxetine or starch (placebo) which were all identical in appearance and weight (50:50). The 100 enrolled patients were given a capsule (containing either 60 mg duloxetine or 60 mg placebo) every night before sleep since preoperative day 2 till postoperative day 14 (17 days in all) by a nurse who were not involved in this trial. Other perioperative managements were the same in the two groups. The primary outcome was the VAS score, including rVAS (visual analogue scale at rest) and aVAS (visual analogue scale upon ambulation) throughout the perioperative period. The secondary outcomes included opioid consumption, range of motion, including both active range of motion (aROM) and passive range of motion (pROM) and adverse events. The patients were followed up everyday until 7 days after TKA, afterwards, they were followed up at the time of 3 weeks and 3 months after TKA.

**Result:**

rVAS in duloxetine group were significantly less than placebo group throughout the postoperative period: 4.7 ± 2.3 vs 5.9 ± 2.6 (*P* = 0.016) at 24 h postoperative; 2.1 ± 1.6 vs 2.8 ± 1.7 (*P* = 0.037) at 7 days postoperative. In terms of aVAS, similarly, duloxetine group had less aVAS than placebo group throughout the postoperative period: 6.2 ± 2.1 vs 7.1 ± 2.2 (*P* = 0.039) at 24 h postoperative; 3.3 ± 1.7 vs 4.1 ± 2.0 (*P* = 0.034) at 7 days postoperative. Patients in duloxetine group consumed significantly less opioids per day than the placebo group: 24.2 ± 10.1 g vs 28.5 ± 8.3 g (*P* = 0.022) at 24 h postoperative; 2.7 ± 2.5 g vs 4.1 ± 2.6 g (*P* = 0.007) at 7 days postoperative. aROM in duloxetine group were significantly better than placebo group until postoperative day 6, the aROM became comparable between the two groups: 110.2 ± 9.9° in duloxetine group vs 107.5 ± 11.5° in control group (*P* = 0.211). In terms of pROM, duloxetine group had significantly better pROM until postoperative day 5, the pROM became comparable between the two groups: 103.8 ± 12.1° in duloxetine group vs 99.5 ± 10.8° in control group (*P* = 0.064). No significant difference was found between the two groups in the rates of dizziness, bleeding, sweating, fatigue and dryness of mouth. In the placebo group, more patients got nausea/vomiting and constipation (*P* < 0.05). However, in terms of drowsiness, duloxetine group was reported higher rate (*P* < 0.05).

**Conclusion:**

Several other RCTs have already mentioned the analgesic effect of duloxetine, but not in the immediate postoperative period. In this study, we found duloxetine could reduce acute postoperative pain in the immediate postoperative period and decrease the opioids consumption as well as accelerating postoperative recovery, without increasing the risk of adverse medication effects in patients undergoing TKA. Duloxetine could act as a good supplement in multimodal pain management protocol for patients undergoing TKA.

**Trial registration statement:**

This study was registered in the Chinese Clinical Trial Registry (ChiCTR2000033910). The date of registration was 06/16/2020.

## Background

Total knee arthroplasty (TKA) is a mature surgery relieving pain as well as improving the function and the life quality of patients, however, it is reported that over 20% of patients undergoing TKA are still dissatisfied, most of which are for the pain [1]. Inadequate pain control would delay rehabilitation and increase the risk of postoperative complications [2]. It is also reported that improper and inadequate postoperative pain management is associated with longer hospital stays, increased rates of unanticipated hospital admissions and readmissions due to pain and an overall increase cost of care [3]. Despite recent advances in multimodal analgesia that reduce opioid consumption, the general use of opioid is still prevalent [2]. Therefore, the current multimodal pain management following TKA still needs optimizing.

Duloxetine (Cymbalta), a potent selective serotonin and norepinephrine dual reuptake inhibitor (SNRI), has been widely approved for major depressive disorder, generalized anxiety disorder, diabetic peripheral neuropathy, fibromyalgia, and chronic musculoskeletal pain [4]. It can increase the levels of both serotonin and norepinephrine by inhibiting the reuptake and finally enhanced the descending inhibitory pain pathways in the central nervous system [5, 6]. Duloxetine’s analgesia effect is independent of its anti-depression effect, it has similar analgesia effects on both depressed and nondepressed patients. Many prior studies have already demonstrated the analgesic effect of duloxetine in patients experiencing chronic musculoskeletal pain such as osteoarthritis knee pain [7], but the peri-TKA use of duloxetine for postoperative pain relief remains controversial: Some studies reported that patients with perioperative use of duloxetine experienced better pain relief after TKA during postoperative weeks 2 to 12, but no apparent benefit was observed in terms of reduced pain or opioid consumption during the immediate postoperative period (weeks 1 and 2) [8], however, some others found that the post-TKA pain of patients with ambulation, flexion, and at rest did not differ between groups throughout postoperative weeks 1 to 6, while the perioperative use of duloxetine could reduce the postoperative daily opioid consumption [9].

Therefore, we performed a prospective randomized, double-blind, placebo-controlled trial in patients undergoing unilateral primary TKA to investigate the analgesic effect of perioperative use of duloxetine. We hypothesized that duloxetine would reduce postoperative pain and morphine consumption relative to placebo, the primary outcome being postoperative VAS scores, including VAS at rest (rVAS) and VAS upon ambulation (aVAS).

## Patients and methods

This prospective randomized, double-blind, placebo-controlled trial study was registered in the Chinese Clinical Trial Registry (ChiCTR2000033910). The date of registration was 06/16/2020. Approval was obtained from the Clinical Trials and Biomedical Ethics Committee of West China Hospital, and written informed consent was obtained from all patients before participation.

### Patients

Patients who were diagnosed with knee osteoarthritis and scheduled to undergo primary unilateral TKA were eligible for study enrollment. Patients were excluded according to the following criteria: American Society of Anesthesiologists physical status IV, a known psychiatric disorder, Hamilton Depression Scale (HAMD) and Hamilton Anxiety Scale (HAMA) scores both < 7, intolerance or allergy to any of the study drugs, alcohol or opioid dependence. Ongoing anticoagulant treatment, preoperative hepatic or renal dysfunction, serious cardiac and/or cerebrovascular comorbidities and refusal to participate. Patients were additionally excluded for concurrent use of duloxetine or other SNRIs, monoamine oxidase inhibitors, tricyclic antidepressants, triptans, lithium, or buspirone.

### Randomization and blinding

Group assignment was concealed from the patients, the nurses, the treating surgeons, the data collectors and the statistician. The hospital pharmacy was provided a random allocation sequence which was computer-generated and concealed in consecutively numbered, opaque, sealed envelopes by a statistician not involved in the data analysis. An experienced surgeon enrolled the patients, and another surgeon reviewed the inclusion criteria and recorded basic information. The hospital pharmacy prepared small capsules containing either duloxetine or starch (placebo) which were all identical in appearance and weight (50:50). All the capsules were given to patients every night before sleep by a nurse who were not involved in this trial.

### Surgical procedure and perioperative management

All the TKAs were performed by the same surgical team. A midline skin incision, a standard medial parapatellar approach and a cemented total knee system-Attune (DePuy, IN, USA) were used in all patients. No tourniquet or drain were applied. Ten milligrams/kg of tranexamic acid (TXA) was given 10 min before skin incision by intravenous infusion, followed by 10 mg/kg administered by local injection when closing the incision; Two doses of 10 mg/kg of TXA were separately administered 3 and 6 h after surgery to reduce perioperative blood loss [10]. Thromboprophylaxis was started in a timely manner: a half dose of low-molecular weight heparin (LMWH, 0.2 mL 2000 IU) was given to patients 6 h postoperatively, and a full dose (0.4 mL 4000 IU) was given at 24 h intervals in the subsequent days by subcutaneous injection till the day of discharge [11]. All the patients follow a standardized physiotherapy program. The criterion of blood transfusion was set as an Hemoglobin level of < 70 or 70 ~ 100 g/L but with symptomatic anemia (defined as severe mental status changes, palpitations, and/or pallor).

### Analgesic protocol and intervention drug

Patients received standardized general anesthesia and basic analgesic protocol. From preoperative day 2 to the day before surgery, patients were given celecoxib 200 mg twice a day (one dose after breakfast, the other one after dinner) for preemptive analgesia [12]. Intraoperatively, all patients received general anesthesia which was induced by sufentanil 0.5 μg/kg, midazolam 0.04 mg/kg, propofol 1–2 mg/kg, and Cisatracurium 2 μg/kg intravenously, followed by continuous intravenous infusion of remifentanil 0.1–0.3 μg/(kg·min), propofol 2–5 mg/(kg·h) and inhalation of sevoflurane to maintain anesthesia. Additionally, an 80-mL periarticular injection of 0.25% ropivacaine was administered to all patients for local infiltration analgesia. Since postoperative day 1, the protocol of oral celecoxib restarted till postoperative 3 weeks when the patients came back to hospital for taking out the stitches. If acute or resistant pain occurred (VAS > 6), the opioids (oral oxycodone or subcutaneous morphine) were used as rescue analgesics.

Patients were given a capsule (containing either 60 mg duloxetine or 60 mg placebo) every night before sleep since preoperative day 2 till postoperative day 14 (17 days in all). The dose of duloxetine, 60 mg, was determined according to the Cochrane database review of duloxetine for painful neuropathy or chronic pain which indicated that the proper dose of oral duloxetine was 60 mg daily, for 20 mg was ineffective and 120 mg was no more effective than 60 mg with more adverse events [4].

### Outcome measurements

The primary outcome was the VAS score (both rVAS and aVAS) throughout the perioperative period, the scale of VAS was set 0 to 10 [4, 7]. The secondary outcomes included opioid consumption, range of motion, including both active ROM (aROM) and passive ROM (pROM) and adverse events.

The VAS score was collected preoperatively and at 2, 4, 6, 24, 36, and 48 h after surgery by a project nurse (2, 4, 6 h after surgery without aVAS), thereafter the average of the VAS in the morning (when getting up) and evening (before going to bed) from postoperative days 2 to 7, 3 weeks and 3 months were also collected. The opioid consumption was recorded every day from the day of surgery to postoperative day 7 by a project nurse, all the opioids no matter oral oxycodone or subcutaneous morphine were converted into morphine equivalent. The ROM was recorded at 6, 24, 36, 48 h after surgery and then from postoperative days 2 to 7, 3 weeks and 3 months in the evening. Finally, adverse events, defined on the basis of previous studies regarding the safety of duloxetine, were recorded since the trial started.

### Statistical analysis

Distributions of demographic data, baseline data, and primary and secondary outcomes were assessed using measures of central tendency (mean, standard deviation) for quantitative variables and with percentages for qualitative variables. The student t test or the Wilcoxon signed-rank test was used to analyze continuous variables, and the chi-square test or the Fisher exact test was used to determine differences in categorical variables. All data analyses were performed using SPSS (version 23.0; IBM). Significance was set at *P* < 0.05.

### Sample-size calculations

The sample-size estimate was based on the primary outcome (VAS pain score). To determine the necessary sample size for sufficient statistical power, we used the results of a previous study on the analgesic effect of duloxetine, which showed that a 1.0-point difference was considered to be clinically relevant [7]. Assuming a common within-group SD of 1.25 points, we calculated that a sample size of 42 patients per group would provide 95% power at a 2-sided α of 0.05 to detect a 1-point difference in mean VAS pain score between the duloxetine and placebo groups using a two-sample t test. Furthermore, the sample size was increased by 20% to compensate for expected dropouts, resulting in 50 patients per group and a total number of 100 patients.

## Result

**Fig. 1:**
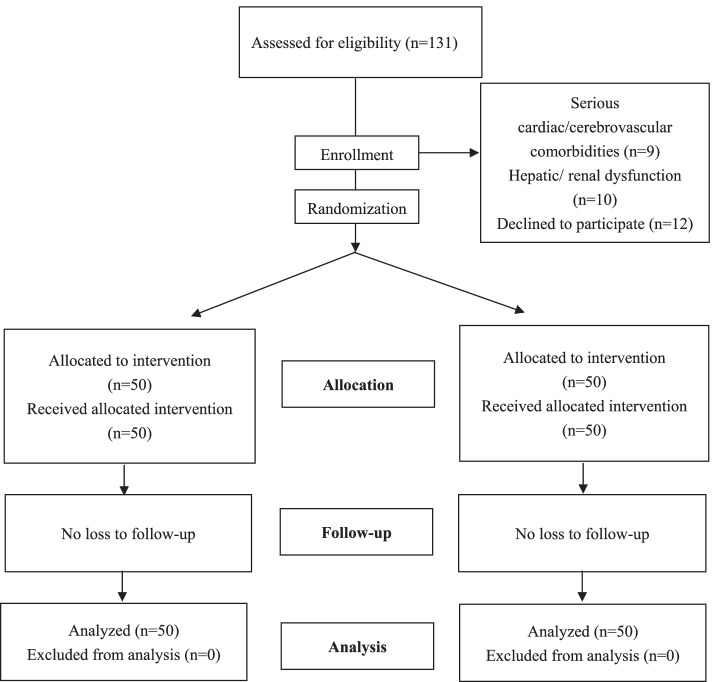
Patients flow chart

From June 8th 2020 to July 31st 2020, 131 patients were scheduled to undergo a total knee arthroplasty at our institution. Among these patients, 19 did not meet the inclusion criteria, 12 declined to participate. Hence, the remaining 100 patients were randomized to two groups (50 patients in each group) (Fig. 1). No patient was lost or excluded during the follow-up period. No significant differences were identified among the groups in patient demographic and preoperative characteristics (Table 1).

**Table 1:** Demographic and preoperative data

	Duloxetine (50)	Placebo (50)	*P*
Age	67.8 ± 10.12	66.2 ± 9.83	0.430
Female	30 (60%)	27 (54%)	0.622
Height	1.58 ± 0.08	1.60 ± 0.09	0.243
Weight	62.97 ± 10.21	63.13 ± 9.92	0.937
BMI	24.67 ± 4.35	24.83 ± 3.87	0.846
rVAS	3.47 ± 1.77	3.62 ± 2.02	0.694
aVAS	6.31 ± 2.35	6.52 ± 2.60	0.673
aROM	100.37 ± 14.21	102.72 ± 13.52	0.399
pROM	104.12 ± 14.69	107.29 ± 14.37	0.278
ASA ІІ/ІІІ	43/7	47/3	0.182
HAMD	3.2 ± 1.1	3.1 ± 1.1	0.650
HAMA	3.1 ± 1.3	3.3 ± 1.2	0.426
Kellgren-Lawrence (K-L) grade III/IV	37/13	36/14	0.822
Preoperative medications			
NSAIDs	12 (24)	15 (30)	0.499
Acetaminophen	2 (4)	2 (4)	1.000
Opioids	0	0	1.000
Antidepressant	0	0	1.000

### Primary outcome

**Fig. 2:**
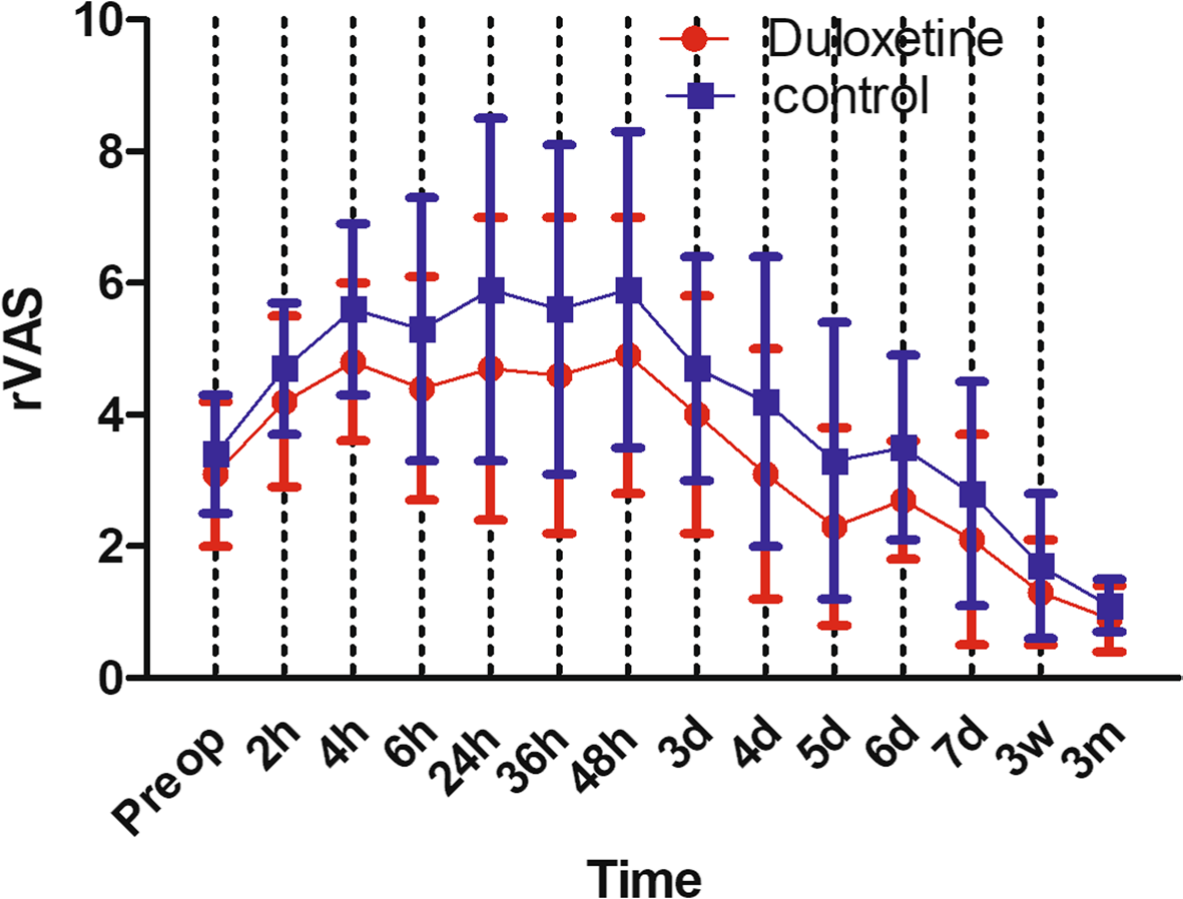
rVAS during the whole study

**Fig. 3:**
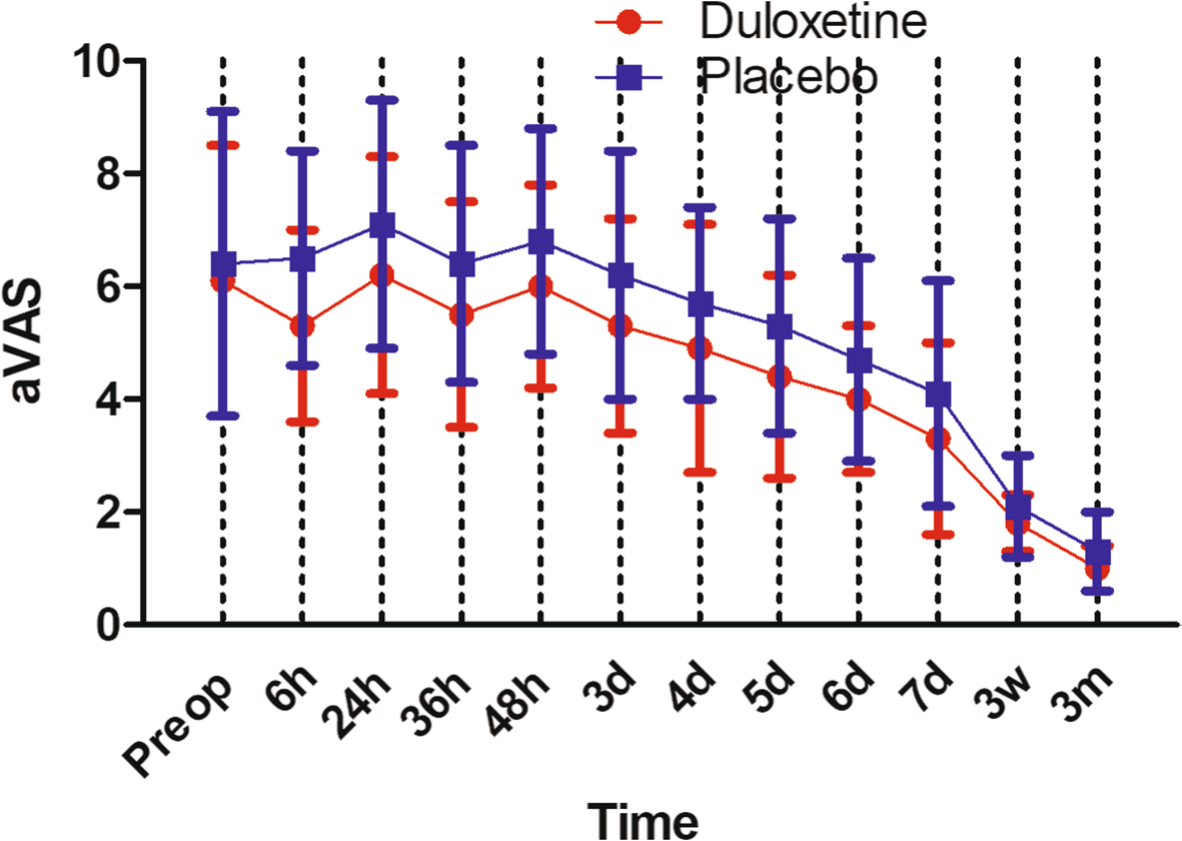
aVAS during the whole study

rVAS in duloxetine group were significantly less than placebo group throughout the postoperative period: 4.7 ± 2.3 vs 5.9 ± 2.6 (*P* = 0.016) at 24 h postoperative; 2.1 ± 1.6 vs 2.8 ± 1.7 (*P* = 0.037) at 7 days postoperative (Fig. 2). In terms of aVAS, similarly, duloxetine group had less aVAS than placebo group throughout the postoperative period: 6.2 ± 2.1 vs 7.1 ± 2.2 (*P* = 0.039) at 24 h postoperative; 3.3 ± 1.7 vs 4.1 ± 2.0 (*P* = 0.034) at 7 days postoperative (Fig. 3).

### Secondary outcomes

**Fig. 4:**
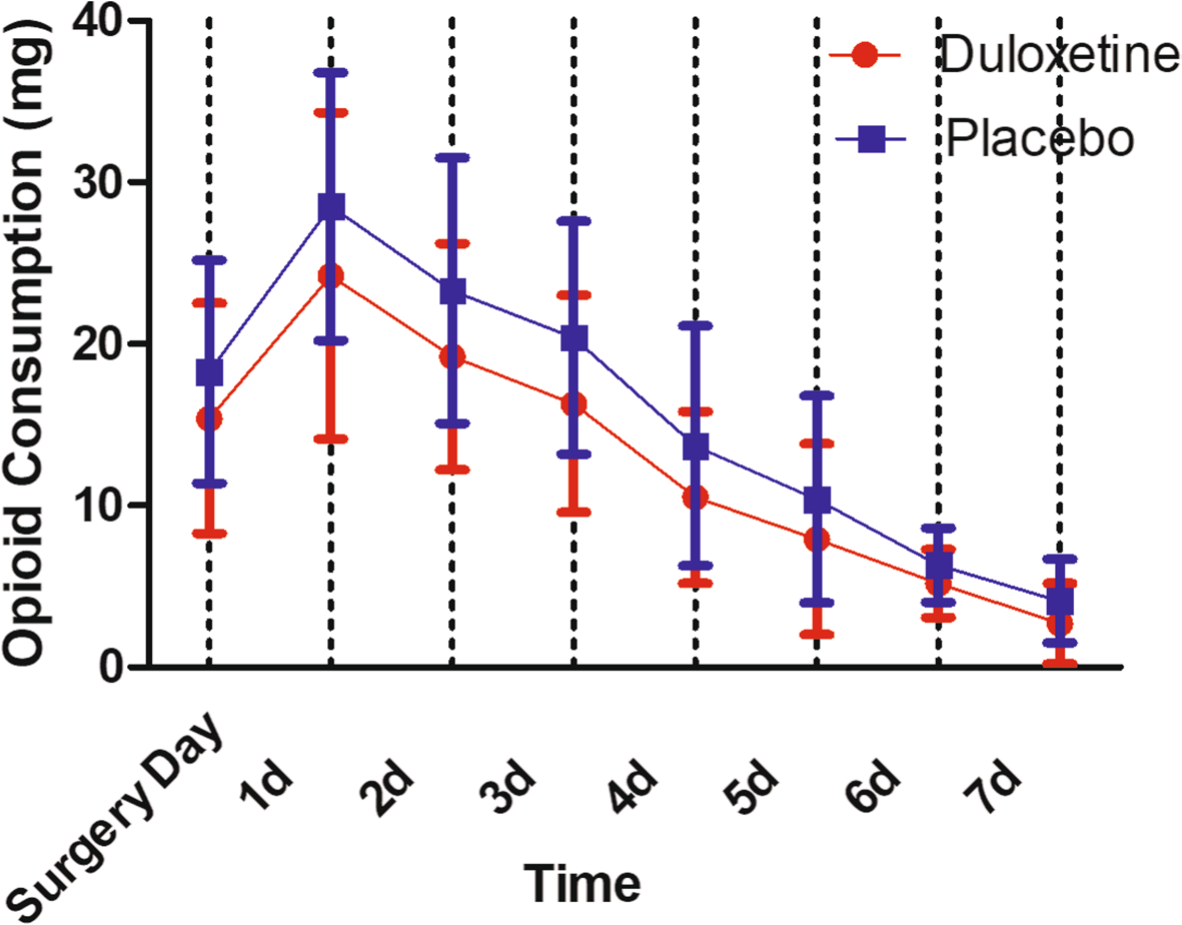
Postoperative opioid consumption

**Fig. 5:**
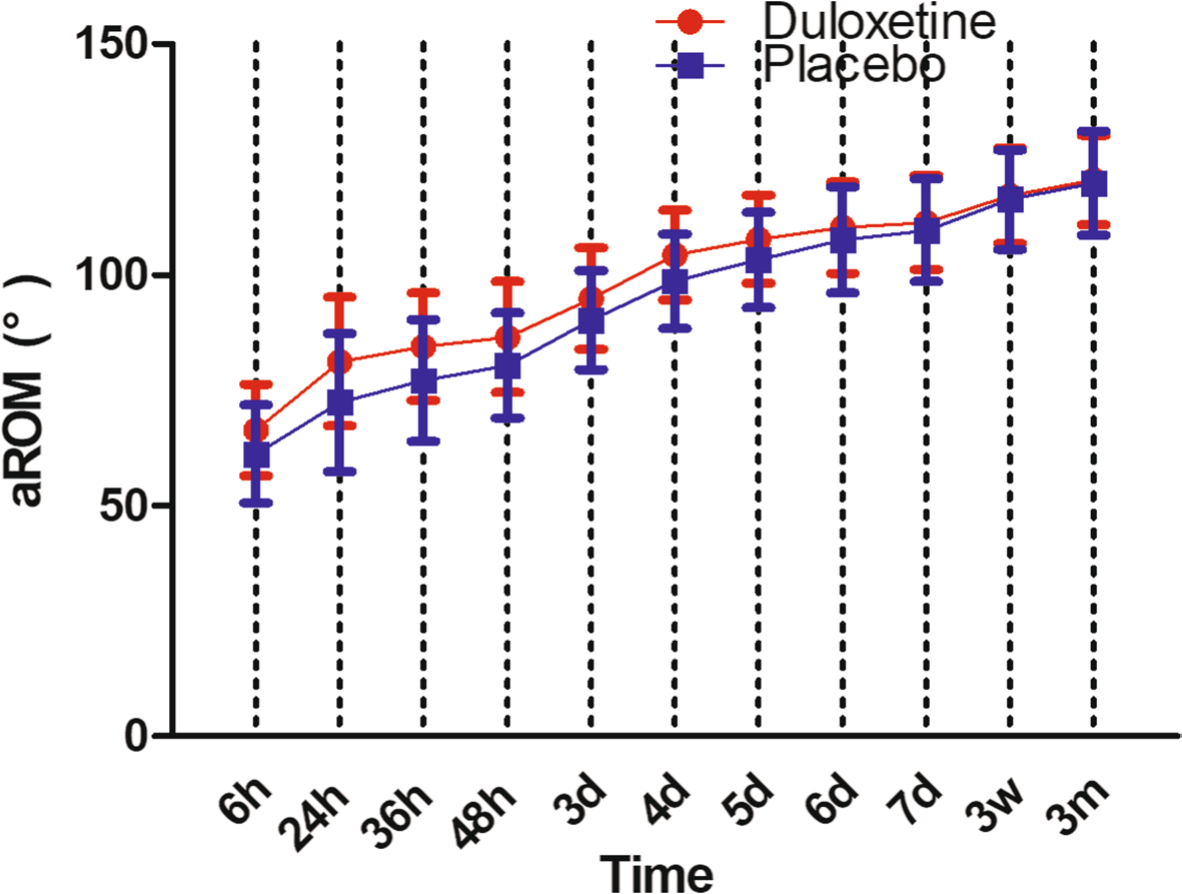
aROM during the whole study

**Fig. 6:**
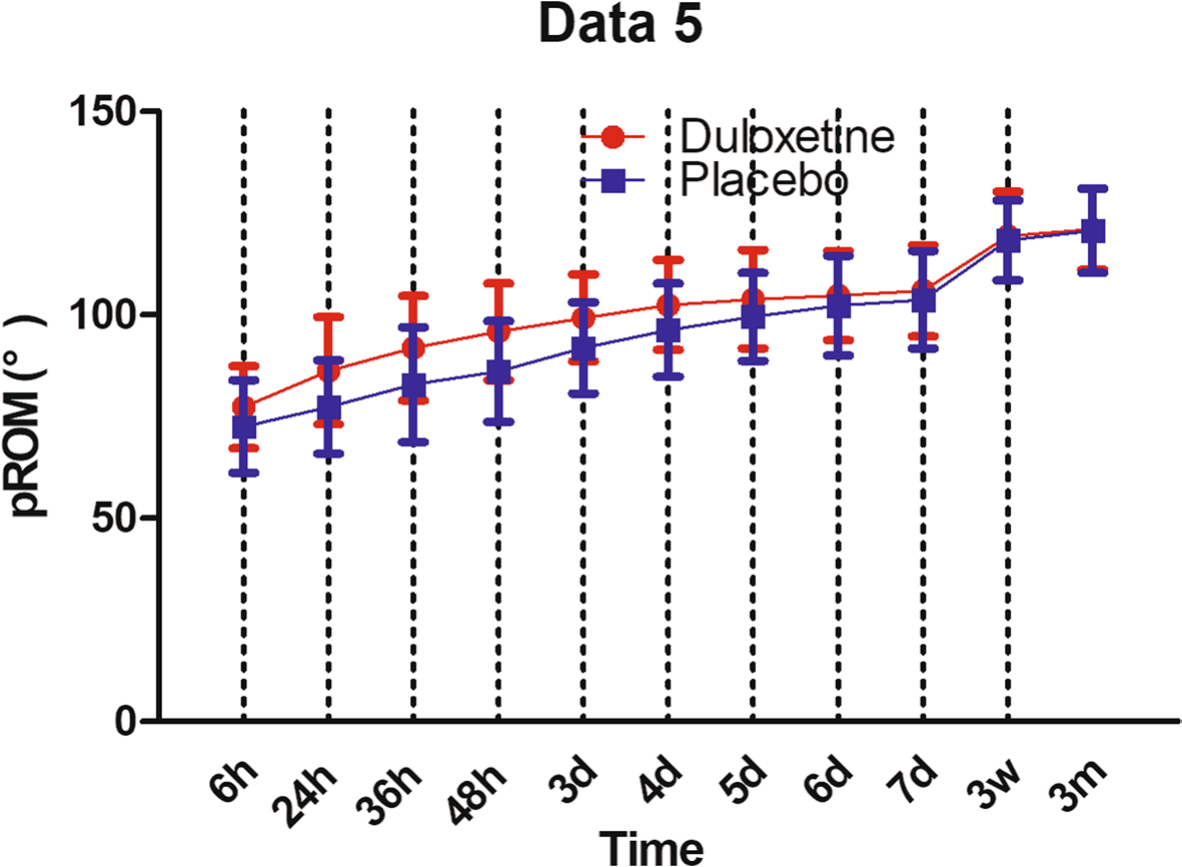
pROM during the whole study

Patients in duloxetine group consumed significantly less opioids per day than the placebo group: 24.2 ± 10.1 g vs 28.5 ± 8.3 g (*P* = 0.022) at 24 h postoperative; 2.7 ± 2.5 g vs 4.1 ± 2.6 g (*P* = 0.007) at 7 days postoperative (Fig. 4). aROM in duloxetine group were significantly better than placebo group until postoperative day 6, the aROM became comparable between the two groups: 110.2 ± 9.9° in duloxetine group vs 107.5 ± 11.5° in control group (*P* = 0.211) (Fig. 5). In terms of pROM, duloxetine group had significantly better pROM until postoperative day 5, the pROM became comparable between the two groups: 103.8 ± 12.1° in duloxetine group vs 99.5 ± 10.8° in control group (*P* = 0.064) (Fig. 6).

Regarding adverse events, no significant difference was found between the two groups in the rates of dizziness, bleeding, sweating, fatigue and dryness of mouth. In the placebo group, more patients got nausea/vomiting and constipation (*P* < 0.05). In terms of drowsiness, duloxetine group was reported higher rate (*P* < 0.05) (Table 2).

**Table 2:** Adverse event

	Duloxetine (*n* = 50)	Placebo (*n* = 50)	*P* value
Nausea/vomiting	5 (10)	15 (30)	0.012
Dizziness	3 (6)	5 (10)	0.461
Drowsiness	20 (40)	10 (20)	0.029
Bleeding	0 (0)	0 (0)	N.A
Sweating	2 (4)	2 (4)	1.000
Fatigue	23 (46)	26 (52)	0.548
Dryness of mouth	18 (36)	17 (34)	0.834
Constipation	3 (6)	11 (22)	0.021

## Discussion

Nowadays, although multimodal analgesia strategies have been widely implemented to control postoperative pain in TKA patients, it was reported that over 20% of patients still suffered from severe postoperative pain [13]. To deal with this problem, surgeons never stopped to explore new methods. Recently, some researchers started to turn their eyes out of regular analgesia drugs, onto the application of antidepressant drugs like tricyclic antidepressants, selective serotonin reuptake inhibitors, and SNRIs, especially duloxetine. The latest review in 2014 which discussed the application of antidepressant drugs for postoperative pain concluded that there was insufficient evidence to support clinical use and called for more high-quality trials to confirm the exact effect of antidepressant drugs on postoperative pain [14]. Duloxetine (Cymbalta), a potent selective SNRI, primarily found to treat depression, has already been demonstrated the analgesic effect in patients with chronic musculoskeletal pain [7]. However, its effect on postoperative pain relief after TKA remains controversial.

So, we conducted this study to detect if duloxetine could be used as an adjuvants or supplement of multimodal analgesia strategies after TKA. In this trial of oral duloxetine 60 mg administered daily for 17 days initiated on preoperative day 2 and continued for 14 days postoperatively, postoperative pain, including both rVAS and aVAS, was significantly reduced relative to placebo through the whole postoperative 7 days. Besides, the placebo group consumed markedly more morphine equivalent during the postoperative period. In terms of secondary outcomes, postoperative recovery of function of the knee (range of motion at different time points) was better in the duloxetine group on the early days after surgery. When it comes to the side effects, patients took duloxetine had better sleep quality and less cases of nausea, vomit or constipation than placebo group, and the difference was marked.

SNRIs, like duloxetine, are targeted presynaptically and postsynaptically on serotonergic and noradrenergic nerve terminals and are the key regulator of synaptic serotonin and noradrenaline levels in central nervous system [15]. The basic function of SNRIs is to block and then down-regulate serotonin and noradrenaline transporters, which therefore increases the concentrations of these two neurotransmitters [16]. The increase of unbound serotonin and noradrenaline would enhance the descending inhibitory pain pathways in the central nervous system [5, 6]. Although the antidepression effect of SNRIs is relatively independent with its analgesia effect, there are still some studies demonstrated that serotonergic signaling in brain regions relevant to affective cognition have been demonstrated to be coupled to tonic pain ratings in healthy volunteers [17, 18]. It was reported that 7–14 days were necessary for SSRIs to take effect in treating anxiety or depression because the brain need time to establish the response [19, 20], so we hypothesized that it might likewise take days for SNRIs to take effect in relieving pain.

Lunn et al. [21] randomized 120 patients scheduled for TKA in a double-blind manner to either 10 mg escitalopram (a kind of SSRI) or placebo daily from pre-anesthesia to postoperative day 6 in addition to a standardized analgesic regime and found that no between-group differences were observed in overall pain upon ambulation from 4 to 48 h or overall pain at rest from 2 to 48 h after surgery. But conversely, overall pain upon ambulation and overall pain at rest from day 2 to 6 after surgery were lower in the escitalopram versus placebo group, which suggested that SSRI could relief postoperative pain but with a delayed attribute. Similarly, Koh et al. [8] randomized 80 patients scheduled for TKA to either duloxetine group (30 mg from the day before surgery and for 6 weeks after surgery) or control group and finally found that patients received duloxetine experienced better pain control during postoperative weeks 2 to 12, while there was no apparent benefit in terms of reduced pain during the immediate postoperative period (weeks 1 and 2). Koh et al. attributed the delayed analgesia to the study design and to the already potent analgesic efficacy of their current multimodal regimen. According to the abovementioned trials, we hypothesized that it might take days as buffer time for duloxetine to take the effect of analgesia. So, we conducted this trial, initiating the intervention of duloxetine much earlier than previous studies (60 mg daily for 17 days initiated on preoperative day 2 and continued for 14 days postoperatively), so that the duloxetine might take effect quickly after surgery, when the pain was supposed to be severe. Consequently, we found that since postoperative 6 h, both the rVAS and aVAS were significantly lower in duloxetine group till postoperative 7 days. It demonstrated that initiating duloxetine early preoperatively could effectively relief the pain of patients after TKA, which was consistent with prior studies and our initial hypothesis.

In terms of the opiate consumption, it has the different function with VAS. Opiate consumption is also a parameter that measures the postoperative pain of patients. We give patients opioids only when the immediate VAS score exceeded 6, which indicates the patient is suffering from severe pain, and the opioids act as a remedial treatment. If we can say the VAS score reflects the patients’ regular condition of pain, we can also say the opiate consumption reflects patients’ fulminant pain. In 2010, Ho et al. [22] first conducted a randomized placebo-controlled trial in 50 patients who received either two doses of oral duloxetine 60 mg (2 h before TKA and on the first day postoperatively) or placebo to specially detect the effect of duloxetine in morphine requirements after TKA and found that the morphine consumption during the 24 h and 48 h after TKA were significantly lower in the duloxetine group, compared with the placebo group. However, the sample size was small in Ho’s study, and only two doses of duloxetine were administrated which could not totally reflect the effect of duloxetine during the postoperative period. Later in 2016, based on the study of Ho et al., YaDeau et al. [9] implemented a longer duration of therapy starting from the day of surgery to the postoperative 14 days (1 dose of 60 mg oral duloxetine daily, 15 doses in all) and found the opioids-reduced effect of duloxetine started quickly after administration. The total daily opioid consumption was significantly less in the duloxetine group from postoperative day 1 to 14, compared with the placebo group, which was consistent with Ho’s study. Similarly, in our study, we prolonged the application of duloxetine before surgery and found that the daily morphine equivalent was markedly less in the duloxetine group from postoperative day 1 to 7, through the early postoperative period. To sum up, the duloxetine could effectively relief the fulminant pain after TKA and this function took effect quickly after application.

Range of motion, which acted as a measurement of recovery of knee function, was significantly better in the duloxetine group during the early postoperative period. Because, both VAS (rVAS and aVAS) and opioid consumption were significantly less in the duloxetine group, and the relatively milder pain would encourage patients in the duloxetine group to be more willing to exercise more frequently and hard, which would therefore accelerate the recovery of the knee function after surgery.

Tolerability and side effects are important issues in analgesic trials. It was reported by Lunn MP et al. [4] that minor adverse effects are common with duloxetine and the regimen of duloxetine 60 mg daily has lower rate of side effects than the dose of 120 mg daily. In our study, although some mild side effects occurred, all the patients were tolerated to the duloxetine regimen of 60 mg per night. Some patients in duloxetine group said they had more drowsiness at night. It is an interesting finding because the drowsiness at night isn’t strictly a side effect, it was reported that a deep sleep at night could relief the nervous mind and unnecessary anxiety. Besides, it was reported by Blagestad et al. [23] that the pain after surgery may obviously contribute to impaired sleep. Oppositely, an improved sleep has also been demonstrated antinociceptive effects [24]. Of note, we noticed that the rates of nausea and vomit as well as constipation were markedly lower in the duloxetine group than in the placebo group. We inferred that the minimum in opioid use by duloxetine might therefore reduce the opioid-related adverse effects especially nausea, vomit and constipation. Although no serious side effects were observed, other studies have suggested an increased risk of perioperative bleeding from the cut and other postoperative morbidities [21]. However, in our study, no such events were observed.

This study had several limitations, firstly, the sample size of this study was calculated according to the primary outcome, so, it might be underpowered to detect other outcomes like rates of various side effects, which calls for future studies to investigate. What’s more, although we initiated the duloxetine intervention earlier than prior studies [9, 22], it still couldn’t be determined whether the duloxetine exerted an effect exactly after surgery, where pain is more pronounced. So, future studies could focus on the optimal duration of perioperative use of duloxetine.

## Conclusion

Several other RCTs have already mentioned the analgesic effect of duloxetine, but not in the immediate postoperative period. In this study, we found duloxetine could reduce acute postoperative pain in the immediate postoperative period and decrease the opioids consumption as well as accelerating postoperative recovery, without increasing the risk of adverse medication effects in patients undergoing TKA. Duloxetine could act as a good supplement in multimodal pain management protocol for patients undergoing TKA.

## Data Availability

Data used and analyzed in this study are available from the corresponding author on reasonable request.

## Abbreviations

TKA: Total knee arthroplasty
BMI: Body mass index
ASA: American Society of Anesthesiologists
rVAS: Visual Analogue Scale at rest
aVAS: Visual Analogue Scale upon ambulation
aROM: Active range of motion
pROM: Passive range of motion
HAMD: Hamilton Depression Scale
HAMA: Hamilton Anxiety Scale
TXA: Tranexamic acid
LMWH: Low-molecular weight heparin
RVM: Rostral ventromedial medulla
